# A ratio chain-type exponential estimator for finite population mean using double sampling

**DOI:** 10.1186/s40064-016-1717-4

**Published:** 2016-01-27

**Authors:** Mursala Khan

**Affiliations:** Department of Mathematics, COMSATS Institute of Information Technology, Abbottabad, 22060 Pakistan; Carl Duisberg Center, Jägerstraße 64, 10117 Mitte-Berlin, Germany

**Keywords:** Double sampling, Study variable, Bias, Auxiliary variable, Mean squared-error, Estimator, Efficiency

## Abstract

In this article, we have proposed a ratio chain-type exponential estimator for finite population mean of the study variable under double sampling scheme using auxiliary variables. The large sample properties of the suggested strategy are derived up to first order, of approximation, and its competence conditions are carried out under which the suggested estimator is performed better than the other existing estimators discussed in the literature. An empirical study shows that the suggested strategy is more efficient than the other relevant competing estimators under two phase sampling scheme.

## Introduction and literature review

To increase the precision of estimators for population mean of the study variable under double sampling design, a lot of works have been done in the field of sample survey and when the study variable is strongly connected with the auxiliary variables the precision of the estimators can be more and more. Using the knowledge of the auxiliary variables several authors have proposed different estimation technique for finite population mean of the study variable, Sukhatme (1962), have developed a general ratio-type estimator. Chand (1975), have suggested two chain ratio-type estimators to estimate the population mean using two auxiliary variables (Kiregyera 1980, 1984; Srivnstava et al. 1990; Bahl and Tuteja 1991; Srivastava 1970; Cochran 1977; Singh et al. 2006, 2007, 2011; Dash and Mishra 2011; Singh and Choudhury 2012; Khare et al. 2013; Khare and Rehman 2013) etc.

Let us consider a finite population of size *N* of different units $U = \left\{ {U_{1} ,U_{2} ,U_{3} , \ldots ,U_{N} } \right\}$. Let *y* and *x* be the study and the auxiliary variable with corresponding values $y_{i}$ and $x_{i}$ respectively for *i-*th unit $i = \left\{ {1,2,3, \ldots ,N} \right\}$ is defined on a finite population *U*.

Let $\bar{Y} = \left( {{1 \mathord{\left/ {\vphantom {1 N}} \right. \kern-0pt} N}} \right)\sum\nolimits_{i = 1}^{N} {y_{i} }$ and $\bar{X} = \left( {{1 \mathord{\left/ {\vphantom {1 N}} \right. \kern-0pt} N}} \right)\sum\nolimits_{i = 1}^{N} {x_{i} }$ be the corresponding population means of the study as well as auxiliary variable respectively. Also let $S_{y}^{2} = \left( {{1 \mathord{\left/ {\vphantom {1 N}} \right. \kern-0pt} N}} \right)\sum\nolimits_{i = 1}^{N} {\left( {y_{i} - \bar{Y}} \right)^{2} }$ and $S_{x}^{2} = \left( {{1 \mathord{\left/ {\vphantom {1 N}} \right. \kern-0pt} N}} \right)\sum\nolimits_{i = 1}^{N} {\left( {x_{i} - \bar{X}} \right)^{2} }$ be the corresponding population variances of the study as well as auxiliary variable respectively and let$C_{y}$ and $C_{x}$ be the coefficient of variation of the study as well as auxiliary variable respectively, and $\rho_{yx}$ be the correlation coefficient between *x* and *y*.

Let *y* and *x* be the study and the auxiliary variable with corresponding values $y_{i}$ and $x_{i}$ respectively for *i-*th unit $i = \left\{ {1,2,3, \ldots ,n} \right\}$ in the sample. Let $\bar{y} = \left( {{1 \mathord{\left/ {\vphantom {1 n}} \right. \kern-0pt} n}} \right)\sum\nolimits_{i = 1}^{n} {y_{i} }$ and $\bar{x} = \left( {{1 \mathord{\left/ {\vphantom {1 n}} \right. \kern-0pt} n}} \right)\sum\nolimits_{i = 1}^{n} {x_{i} }$ be the corresponding unbiased sample means of the study as well as auxiliary variable respectively.

Also let $s_{y}^{2} = \left( {{1 \mathord{\left/ {\vphantom {1 {n - 1}}} \right. \kern-0pt} {n - 1}}} \right)\sum\nolimits_{i = 1}^{n} {\left( {y_{i} - \bar{y}} \right)^{2} }$ and $s_{x}^{2} = \left( {{1 \mathord{\left/ {\vphantom {1 {n - 1}}} \right. \kern-0pt} {n - 1}}} \right)\sum\nolimits_{i = 1}^{n} {\left( {x_{i} - \bar{x}} \right)^{2} }$ be the corresponding unbiased sample variances of the study as well as auxiliary variable respectively.

Let $S_{yx}$, $S_{yz}$ and $S_{xz}$ be the co-variances between their respective subscripts respectively. Similarly $b_{yx} = \frac{{s_{yx} }}{{s_{x}^{2} }}$ is the corresponding sample regression coefficient *y* on *x* based on a sample of size *n*. Also $C_{y} = \frac{{S_{y} }}{{\bar{Y}}},\;C_{x} = \frac{{S_{x} }}{{\bar{X}}}$ and $C_{z} = \frac{{S_{z} }}{{\bar{Z}}}$ are the coefficients of variation of the study and auxiliary variables respectively.

The usual unbiased estimator to estimate the population mean of the study variable is

1 $$ \bar{y}_{0} = \frac{1}{n}\sum\limits_{i = 1}^{n} {y_{i} } $$

The variance of the estimator $\bar{y}$ up to first order of approximation is, given by

2 $$ V\left( {\bar{y}_{0} } \right) = f_{1} \bar{Y}^{2} C_{y}^{2} $$

The usual ratio and regression estimators in two phase sampling and their mean square error are, given as follows

3 $$ \bar{y}_{1} = \frac{{\bar{y}}}{{\bar{x}}}\bar{x}^{\prime} $$

4 $$ \bar{y}_{2} = \bar{y} + b_{yx} \left( {\bar{x}^{\prime} - \bar{x}} \right) $$

The mean squared error and variance are given below

5 $$ MSE\left( {\bar{y}_{1} } \right) = \bar{Y}^{2} \left[ {f_{1} C_{y}^{2} + f_{3} \left( {C_{x}^{2} - 2\rho_{yx} C_{y} C_{x} } \right)} \right] $$

6 $$ Var\left( {\bar{y}_{2} } \right) = S_{y}^{2} \left[ {f_{1} \left( {1 - \rho_{yx}^{2} } \right) + f_{2} \rho_{yx}^{2} } \right] $$

where $$ f_{1} = \left( {\frac{1}{n} - \frac{1}{N}} \right),\quad f_{2} = \left( {\frac{1}{{n^{\prime}}} - \frac{1}{N}} \right) $$ and $$ \, f_{3} = \left( {\frac{1}{n} - \frac{1}{{n^{\prime}}}} \right). $$

Chand (1975), proposed the following chain ratio-type estimator in double sampling by incorporating the knowledge of two auxiliary variables, the suggested estimator is, given by

7 $$ \bar{y}_{3} = \frac{{\bar{y}}}{{\bar{x}}}\frac{{\bar{x}^{\prime}}}{{\bar{z}^{\prime}}}\bar{Z} $$

The mean square error of the suggested estimator is, given as

8 $$ MSE\left( {\bar{y}_{3} } \right) = \bar{Y}^{2} \left[ \begin{aligned}& f_{1} C_{y}^{2} + f_{3} \left( {C_{x}^{2} - 2\rho_{yx} C_{y} C_{x} } \right) \hfill \\& + f_{2} \left( {C_{z}^{2} - 2\rho_{yz} C_{y} C_{z} } \right)  \\ \end{aligned} \right] $$

Kiregyera (1984), suggested the following chain-type exponential estimators in two phase sampling, the suggested estimators are given as

9 $$ \bar{y}_{4} = \frac{{\bar{y}}}{{\bar{x}}}\left[ {\bar{x}^{\prime} + b_{xz} \left( {\bar{Z} - \bar{z}^{\prime}} \right)} \right] $$

10 $$ \bar{y}_{5} = \bar{y} + b_{yx} \left[ {\left( {\bar{x}^{\prime} - \bar{x}} \right) - b_{xz} \left( {\bar{Z} - \bar{z}^{\prime}} \right)} \right] $$

The mean square errors of the suggested estimators, up to first order of approximation are, given as follows

11 $$ MSE\left( {\bar{y}_{4} } \right) = \bar{Y}^{2} \left[ \begin{aligned} f_{1} C_{y}^{2} + f_{3} C_{x} \left( {C_{x} - 2\rho_{yx} C_{y} } \right) \hfill \\ + f_{2} \rho_{xz} C_{x} \left( {\rho_{xz} C_{x} - 2\rho_{yz} C_{y} } \right) \hfill \\ \end{aligned} \right] $$

12 $$ MSE\left( {\bar{y}_{5} } \right) = \bar{Y}^{2} C_{y}^{2} \left[ \begin{aligned} &f_{2} \rho_{yx} \rho_{xz} \left( {\rho_{yx} \rho_{xz} - 2\rho_{yz} } \right) \hfill \\& + f_{1} - f_{3} \rho_{yx}^{2} \hfill \\ \end{aligned} \right] $$

Searls (1964), proposed an estimation procedure for population mean using known knowledge of the coefficient of variation of the auxiliary variable

13 $$ \bar{y}^{ * } = a\bar{y} $$

14 $$ \text{var} (\bar{y}^{ * } ) = \left( {1 - B} \right)f_{1} \bar{Y}^{2} C_{y}^{2} $$

where $$ a = \left\{ {1 + f_{1} \bar{Y}^{2} C_{y}^{2} } \right\}^{ - 1} $$ and $$ B = f_{1} \bar{Y}^{2} C_{y}^{2} $$

Khare and Rehman (2013), have proposed improved chain type estimators for population mean using auxiliary information, the suggested estimators are given by

15 $$ \bar{y}_{6} = \frac{{\bar{y}^{ * } }}{{\bar{x}}}\left[ {\bar{x}^{\prime} + b\left( {\bar{Z} - \bar{z}^{\prime}} \right)} \right] $$

16 $$ \bar{y}_{7} = \bar{y}^{ * } + b_{1} \left[ {\left( {\bar{x}^{\prime} - \bar{x}} \right) - b_{2} \left( {\bar{Z} - \bar{z}^{\prime}} \right)} \right] $$

where $b$, $b_{1}$ and $b_{2}$ are constants.

The mean square errors of the suggested estimators, are, given by

17 $$ MSE\left( {\bar{y}_{6} } \right) = \bar{Y}^{2} \left[ \begin{aligned}& + b^{2} R^{2} f_{2} C_{z}^{2} - 2bR\left( {1 - B} \right)f_{2} \rho_{yz} C_{y} C_{z} \hfill \\ &+ f_{3} C_{x} \left\{ {C_{x} - 2\left( {1 - B} \right)\rho_{yx} C_{y} } \right\} \hfill \\ &+ \left( {1 - B} \right)f_{1} C_{y}^{2} \hfill \\ \end{aligned} \right] $$

and

18 $$ MSE\left( {\bar{y}_{7} } \right) = \left[ \begin{aligned}& b_{1} f_{3} \bar{X}C_{x} \left( {b_{1} \bar{X}C_{x} - 2\left( {1 - B} \right)\bar{Y}\rho_{yx} C_{y} } \right) \hfill \\ &+ b_{1} b_{2} \bar{Z}f_{2} C_{z} \left( {b_{1} b_{2} \bar{Z}C_{z} - 2\bar{Y}\left( {1 - B} \right)\rho_{yz} C_{y} } \right) \hfill \\ &+ \bar{Y}^{2} \left( {1 - B} \right)f_{1} C_{y}^{2} \hfill \\ \end{aligned} \right] $$

where the optimum values of $b$, $b_{1}$ and $b_{2}$ are $b_{opt} = \frac{{\left( {1 - B} \right)C_{yz} }}{{RC_{z}^{2} }},\;b_{1opt} = \frac{{ - k_{2} \pm \sqrt {k_{2}^{2} - 4k_{1} k_{3} } }}{{2k_{1} }}$ and $b_{2opt} = \frac{M}{{b_{1opt} }}$.

Also $$ M = \frac{{\bar{Y}\left( {1 - B} \right)C_{yz} }}{{\bar{Z}C_{z}^{2} }}, $$$$ k_{1} = f_{3} b_{1}^{2} \bar{X}^{2} C_{x}^{2} $$, $$ k_{2} = f_{3} \left( {B - 1} \right)\bar{Y}\bar{X}b_{1} C_{yx} , $$$$ R = {{\bar{Z}} \mathord{\left/ {\vphantom {{\bar{Z}} {\bar{X}}}} \right. \kern-0pt} {\bar{X}}} $$ and $$ k_{2} = M\bar{Z}f_{2} C_{z} \left\{ {M\bar{Z}C_{z} - \bar{Y}\left( {1 - B} \right)\rho_{yz} C_{y} } \right\}. $$

Singh et al. (2013), recommended a class of exponential chain ratio-product type estimator for estimating population mean using two auxiliary variables, as

19 $$ \bar{y}_{7} = \bar{y}\left\{ {\alpha \exp \left( {\frac{{\bar{x}^{\prime}\frac{{\bar{Z}}}{{\bar{z}^{\prime}}} - \bar{x}}}{{\bar{x}^{\prime}\frac{{\bar{Z}}}{{\bar{z}^{\prime}}} + \bar{x}}}} \right) + \beta \exp \left( {\frac{{\bar{x} - \bar{x}^{\prime}\frac{{\bar{Z}}}{{\bar{z}^{\prime}}}}}{{\bar{x} + \bar{x}^{\prime}\frac{{\bar{Z}}}{{\bar{z}^{\prime}}}}}} \right)} \right\} $$

where $\alpha$ and $\beta$ are suitably chosen constants, such that $\alpha + \beta = 1$

The minimum mean square error of the suggested estimator is given as follows

20 $$ MSE\left( {\bar{y}_{8} } \right) = \bar{Y}^{2} C_{y}^{2} \left[ {f_{1} - \frac{{\left( {\rho_{yx} f_{3} C_{x} + \rho_{yz} f_{2} C_{z} } \right)^{2} }}{{\left( {f_{3} C_{x}^{2} + f_{2} C_{z}^{2} } \right)}}} \right] $$

where the optimum value of $\alpha$ is $\alpha_{opt} = \frac{1}{2} + \frac{{\left( {\rho_{yx} f_{3} C_{xy} + \rho_{yz} f_{2} C_{zy} } \right)}}{{\left( {f_{3} C_{x}^{2} + f_{2} C_{z}^{2} } \right)}}$.

## The proposed estimator

Let us consider a finite population $U = \left\{ {U_{1} ,U_{2} ,U_{3} , \ldots ,U_{N} } \right\}$ of size *N* units. To estimate the population mean $\bar{Y}$ of the variable of interest *y* taking values $y_{i}$, in the existence of two auxiliary variables say *x* and *z* taking values $x_{i}$ and $z_{i}$ respectively for the *i*th unit $U_{i}$. We assume that there is high correlation between *y* and *x* as compared to the correlation between *y* and *z* (i.e. $\rho_{yx} > \rho_{yz} > 0$). When the population $\bar{X}$ of the auxiliary variable *x* is unknown, but information on the other cheaply auxiliary variable *z* closely related to *x* but compared to *x* remotely to *y*, is available for all the units in a population. In such a situation we use two phase sampling. In the two phase sampling scheme a large initial sample of size $n^{\prime}(n^{\prime} < N)$ is drawn from the population $U$ by using simple random sample without replacement sampling (SRSWOR) scheme and measure *x* and *z* to estimate $\bar{X}$. In the second phase, we draw a sample (subsample) of size $n$ from first phase sample of size $n^{\prime}$, i.e. ($n < n^{\prime}$) by using SRSWOR or directly from the population *U* and observed the study variable *y*.

Under the given probability sampling, we have proposed a chain-ratio-type exponential estimator for finite population mean of the study variable *y*, given by

21 $$ \bar{y}_{m} = \bar{y}\left( {\frac{{\bar{x}^{\prime} - \bar{x}}}{{\bar{x}^{\prime} + \bar{x}}}} \right)^{{k_{1} }} + k_{2} \left\{ {\bar{x}^{\prime}\exp \left( {\frac{{\bar{Z} - \bar{z}^{\prime}}}{{\bar{Z} + \bar{z}^{\prime}}}} \right) - \bar{x}} \right\} $$

where $k_{1}$ and $k_{2}$ are the unknown constants, whose value is to be determined for optimality conditions.

To obtain the properties of the proposed estimator we define the following relative error terms and their expectations.

Let $$ e_{0} = \frac{{\bar{y} - \bar{Y}}}{{\bar{Y}}},e_{1} = \frac{{\bar{x} - \bar{X}}}{{\bar{X}}},e^{\prime}_{1} = \frac{{\bar{x}^{\prime} - \bar{X}}}{{\bar{X}}},e_{2} = \frac{{\bar{z} - \bar{Z}}}{{\bar{Z}}} $$ and $$ e^{\prime}_{2} = \frac{{\bar{z} - \bar{Z}}}{{\bar{Z}}}. $$

Such that $E\left( {e_{i} } \right) = E\left( {e^{\prime}_{i} } \right) = 0.$ For *i* = 0, 1, 2.

$$ E\left( {e_{0}^{2} } \right) = f_{1} C_{y}^{2} ,E\left( {e_{1}^{2} } \right) = f_{1} C_{x}^{2} ,E\left( {e_{2}^{2} } \right) = f_{1} C_{z}^{2} , $$

$$ E\left( {e_{1}^{\prime 2} } \right) = E\left( {e_{1} e^{\prime}_{1} } \right) = f_{2} C_{x}^{2} ,E\left( {e_{0} e^{\prime}_{2} } \right) = f_{2} C_{yz} , $$

$$ E\left( {e_{0} e_{1} } \right) = f_{1} C_{yx} ,\;E\left( {e_{0} e^{\prime}_{1} } \right) = f_{2} C_{yx} ,\;E\left( {e_{0} e_{2} } \right) = f_{1} C_{yz} , $$

$$ E\left( {e_{1} e^{\prime}_{2} } \right) = E\left( {e^{\prime}_{1} e_{2} } \right) = E\left( {e^{\prime}_{1} e^{\prime}_{2} } \right) = f_{2} C_{xz} , $$

$$ E\left( {e_{2}^{{{\prime }2}} } \right) = E\left( {e_{2} e^{\prime}_{2} } \right) = f_{2} C_{z}^{2} ,\;E\left( {e_{1} e_{2} } \right) = f_{1} C_{xz} . $$

Equation (21) can be further simplified as given by

22 $$ \bar{y}_{m} = \bar{Y}\left( {1 + e_{0} } \right)\left( {\frac{{e^{\prime}_{1} - e_{1} }}{{2 + e^{\prime}_{1} + e_{1} }}} \right)^{{K_{1} }} + k_{2} \bar{X}\left\{ {\left( {1 + e^{\prime}_{1} } \right)\left( {1 - \frac{{e^{\prime}_{2} }}{2} + \frac{{3e_{2}^{{{\prime }2}} }}{8}} \right) - \left( {1 + e_{1} } \right)} \right\} $$

Further simplifying, up to order one

23 $$ \bar{y}_{m} = \bar{Y}\left\{ \begin{aligned} &1 + e_{0} + \frac{{k_{1} \left( {e^{\prime}_{1} - e_{1} } \right)}}{2} - \frac{{k_{1} \left( {e^{\prime}_{1} + e_{1} } \right)\left( {e^{\prime}_{1} - e_{1} } \right)}}{4} \hfill \\& + \frac{{k_{1} \left( {k_{1} - 1} \right)\left( {e^{\prime}_{1} - e_{1} } \right)^{2} }}{4} + \frac{{k_{1} e_{0} \left( {e^{\prime}_{1} - e_{1} } \right)}}{2} \hfill \\ \end{aligned} \right\} +\, k_{2} \bar{X}\left( {e^{\prime}_{1} - \frac{{e^{\prime}_{2} }}{2} - \frac{{e^{\prime}_{1} e^{\prime}_{2} }}{2} + \frac{{3e_{2}^{{{\prime }2}} }}{8}} \right) $$

Expanding the right hand side of the Eq. (23), up to first order of approximation and subtracting $\bar{Y}$ from both sides we get

24 $$ \bar{y}_{m} - \bar{Y} = \bar{Y}\left\{ \begin{aligned} e_{0} + \frac{{k_{1} \left( {e^{\prime}_{1} - e_{1} } \right)}}{2} - \frac{{k_{1} \left( {e^{\prime}_{1} + e_{1} } \right)\left( {e^{\prime}_{1} - e_{1} } \right)}}{4} \hfill \\ + \frac{{k_{1} \left( {k_{1} - 1} \right)\left( {e^{\prime}_{1} - e_{1} } \right)^{2} }}{4} + \frac{{k_{1} e_{0} \left( {e^{\prime}_{1} - e_{1} } \right)}}{2} \hfill \\ \end{aligned} \right\} + \,k_{2} \bar{X}\left( {e^{\prime}_{1} - \frac{{e^{\prime}_{2} }}{2} - \frac{{e^{\prime}_{1} e^{\prime}_{2} }}{2} + \frac{{3e_{2}^{{{\prime }2}} }}{8}} \right) $$

On squaring and taking expectation on both sides of (24), we get mean square error up to first order of approximation, given as

25 $$ MSE\left( {\bar{y}_{m} } \right) = \bar{Y}^{2} C_{y}^{2} \left[ \begin{aligned} &\bar{Y}^{2} f_{1} C_{y}^{2} - \bar{Y}^{2} k_{1} f_{3} C_{yx} + \bar{Y}\bar{X}k_{2} f_{2} \left( {2C_{yx} - C_{yz} } \right) \hfill \\ &+ \frac{1}{4}k_{2}^{2} \bar{X}^{2} f_{2} \left( {C_{z}^{2} + 4C_{x}^{2} - 4C_{xz} } \right) + \frac{1}{4}k_{1}^{2} \bar{Y}^{2} f_{3} C_{x}^{2} \hfill \\ \end{aligned} \right] $$

Differentiating Eq. (25) w.r.t to $k_{1}$ and $k_{2}$ we get the optimum values of $k_{1}$ and $k_{2}$ respectively as given by, where the optimum values are $k_{1opt} = \frac{{2\rho_{xy} C_{y} }}{{C_{x} }}$ and $k_{2opt} = \frac{{ - 2\bar{Y}\left( {2C_{yx} - C_{yz} } \right)}}{{\bar{X}\left( {4C_{x}^{2} + C_{z}^{2} - 4C_{xz} } \right)}}$ respectively.

On substituting the optimum value of $k_{1}$ and $k_{2}$ in Eq. (25), we get the minimum mean square error of the proposed estimators, given as follows

26 $$ MSE\left( {\bar{y}_{m} } \right)_{\hbox{min} } = \bar{Y}^{2} C_{y}^{2} \left[ {f_{1} - f_{3} \rho_{xy}^{2} - \frac{{f_{2} \left( {2\rho_{xy} C_{x} - \rho_{yz} C_{z} } \right)^{2} }}{{\left( {4C_{x} \left( {C_{x} - \rho_{xz} C_{z} } \right) + C_{z}^{2} } \right)}}} \right] $$

## Efficiency comparison

In this section, we have obtained some conditions by comparing the mean square errors of the estimators under which the proposed estimator performs better than the other existing estimators. The proposed estimator $\bar{y}_{m}$ is more efficient if the given conditions are satisfied.

(i) By (26) and (2), $MSE\left( {\bar{y}_{m} } \right)_{\hbox{min} } \le MSE\left( {\bar{y}_{0} } \right)$ if,
  $$ \left[ {f_{3} \rho_{xy}^{2} + \frac{{f_{2} \left( {2\rho_{yx} C_{x} - \rho_{yz} C_{z} } \right)^{2} }}{{\left( {4C_{x}^{2} + C_{z}^{2} - 4C_{xz} } \right)}}} \right] \ge 0. $$
(ii) By (26) and (5), $MSE\left( {\bar{y}_{m} } \right)_{\hbox{min} } \le MSE\left( {\bar{y}_{1} } \right)$ if,
  $$ \left[ \begin{aligned} \left( {C_{x}^{2} + \rho_{xy}^{2} C_{y}^{2} - 2\rho_{yx} C_{y} C_{x} } \right) \hfill \\ + \frac{{C_{y}^{2} f_{2} \left( {2\rho_{xy} C_{x} - \rho_{yz} C_{z} } \right)^{2} }}{{\left( {4C_{x} \left( {C_{x} - \rho_{xz} C_{z} } \right) + C_{z}^{2} } \right)}} \hfill \\ \end{aligned} \right] \ge 0. $$
(iii) By (26) and (6), $MSE\left( {\bar{y}_{m} } \right)_{\hbox{min} } \le Var\left( {\bar{y}_{2} } \right)$ if,
  $$ \left[ {\frac{{f_{2} \left( {2\rho_{yx} C_{x} - \rho_{yz} C_{z} } \right)^{2} }}{{\left( {4C_{x}^{2} + C_{z}^{2} - 4C_{xz} } \right)}}} \right] \ge 0. $$
(iv) By (26) and (8), $MSE\left( {\bar{y}_{m} } \right)_{\hbox{min} } \le MSE\left( {\bar{y}_{3} } \right)$ if,
  $$ \left[ \begin{aligned} &f_{3} \left( {C_{y}^{2} \rho_{xy}^{2} + C_{x}^{2} - 2C_{yx} } \right)  \hfill \\& +f_{2} \left( {\frac{{\left( {2C_{xy} - C_{yz} } \right)^{2} }}{{\left( {4C_{x}^{2} + C_{z}^{2} - 4C_{xz} } \right)}} + \left( {C_{z}^{2} - 2C_{yz} } \right)} \right) \hfill \\ \end{aligned} \right] \ge 0. $$
(v) By (26) and (11), $MSE\left( {\bar{y}_{m} } \right)_{\hbox{min} } \le MSE\left( {\bar{y}_{4} } \right)$ if,
  $$ \left[ \begin{aligned}& f_{3} \left( {C_{y}^{2} \rho_{xy}^{2} + C_{x}^{2} - 2C_{yx} } \right)  \hfill \\ &+f_{2} \left( \begin{aligned} \frac{{\left( {2C_{xy} - C_{yz} } \right)^{2} }}{{\left( {4C_{x}^{2} + C_{z}^{2} - 4C_{xz} } \right)}} + \hfill \\ C_{x} \rho_{xz} \left( {C_{x} \rho_{xz} - 2\rho_{yz} C_{y} } \right) \hfill \\ \end{aligned} \right) \hfill \\ \end{aligned} \right] \ge 0. $$
(vi) By (26) and (12), $MSE\left( {\bar{y}_{m} } \right)_{\hbox{min} } \le MSE\left( {\bar{y}_{5} } \right)$ if,
  $$ \left[ {\rho_{yx} \rho_{xz} \left( {\rho_{yx} \rho_{xz} - 2\rho_{yz} } \right) + \frac{{\left( {2\rho_{xy} C_{x} - \rho_{yz} C_{z} } \right)^{2} }}{{\left( {4\left( {C_{x}^{2} - C_{xz} } \right) + C_{z}^{2} } \right)}}} \right] \ge 0. $$
(vii) By (26) and (17), $MSE\left( {\bar{y}_{m} } \right)_{\hbox{min} } \le MSE\left( {\bar{y}_{6} } \right)$ if,
  $$ \left[ \begin{aligned} &f_{3} \left( {C_{y}^{2} \rho_{xy}^{2} + C_{x}^{2} - 2\left( {1 - B} \right)C_{yx} } \right) - Bf_{1} C_{y}^{2} \hfill \\ &+ f_{2} \left\{ \begin{aligned} &\frac{{\left( {2C_{xy} - C_{yz} } \right)^{2} }}{{\left( {4C_{x}^{2} + C_{z}^{2} - 4C_{xz} } \right)}}  \hfill \\ &+b^{2} R^{2} C_{z}^{2} - 2bR\left( {1 - B} \right)C_{yz} \hfill \\ \end{aligned} \right\} \hfill \\ \end{aligned} \right] \ge 0. $$
(viii) By (26) and (18), $MSE\left( {\bar{y}_{m} } \right)_{\hbox{min} } \le MSE\left( {\bar{y}_{7} } \right)$ if,
  $$ \left[ \begin{aligned} &f_{3} \left\{ {S_{y}^{2} \rho_{xy}^{2} + b_{1}^{2} S_{x}^{2} - 2\left( {1 - B} \right)b_{1} S_{yx} } \right\} - \left( {f_{1} S_{y}^{2} } \right)^{2} \hfill \\ &+ f_{2} \left\{ \begin{aligned} &\frac{{\bar{Y}^{2} \left( {2C_{xy} - C_{yz} } \right)^{2} }}{{\left( {4C_{x}^{2} + C_{z}^{2} - 4C_{xz} } \right)}} \hfill \\& + b_{1} b_{2} \left( {b_{1} b_{2} \bar{Z}S_{z}^{2} - 2\left( {1 - B} \right)S_{yz} } \right) \hfill \\ \end{aligned} \right\} \hfill \\ \end{aligned} \right] \ge 0. $$
(ix) By (26) and (20), $MSE\left( {\bar{y}_{m} } \right)_{\hbox{min} } \le MSE\left( {\bar{y}_{8} } \right)$ if,
  $$ \left[ \begin{aligned} &f_{3} \rho_{xy}^{2} + \frac{{f_{2} \left( {2\rho_{xy} C_{x} - \rho_{yz} C_{z} } \right)^{2} }}{{\left( {4C_{x} \left( {C_{x} - \rho_{xz} C_{z} } \right) + C_{z}^{2} } \right)}} - \hfill \\ &\frac{{\left( {\rho_{yx} f_{3} C_{x} + \rho_{yz} f_{2} C_{z} } \right)^{2} }}{{\left( {f_{3} C_{x}^{2} + f_{2} C_{z}^{2} } \right)}} \hfill \\ \end{aligned} \right] \ge 0. $$

## Numerical study

To verify the theoretical conditions under the efficiency comparison numerically, we have taken a real data set from the literature. The description and the necessary data statistics of the data is, given below.

For percent relative efficiency we use the following formula.

$$ PRE = \left[ {\frac{{Var\left( {\bar{y}_{0} } \right)}}{{MSE\left( {\bar{y}_{j} } \right)}}} \right] * 100, $$

for *j* = 1, 2, 3, 4, 5, 6, 7, 8 and *m*.

### Population

The data from the population of 100 records of resale of homes from February 15 to April 30, 1993 from the files maintained by the Albuquerque Board of realtors on selling price ($) as a study variable *y* square feet of living space as an auxiliary variable *x* and annual taxes ($) as an additional variable *z* have been taken. The numerical values of the parameters of the population are given as.

$$ \bar{Y} = 1093.41,\quad \bar{X} = 1697.44,\quad \bar{Z} = 801.58, $$

$$ C_{y}^{2} = 0.1285,\quad \;C_{x}^{2} = 0.0994,\quad C_{z}^{2} = 0.1560, $$

$$ S_{y} = 391.90,\quad \;S_{x} = 535.01,\quad S_{z} = 316.62, $$

$$ \rho_{yx} = 0.84,\quad \rho_{yz} = 0.64,\quad \;\rho_{xz} = 0.86. $$

## Conclusion

On the basis of mean square errors and the percent relative efficiencies of the estimators as shown in Tables 1 and 2, it has been observed that the performance of the proposed estimator is better than the other relevant existing estimators discussed in the literature of survey sampling, which reveals the usefulness of suggested method in practice and would work very well in practical surveys.

**Table 1:** Mean square errors (*MSE’s*) of the estimators

Estimators	Mean squared errors (MSE’s)		
	n′ = 60, n = 30	n′ = 65, n = 35	n′ = 70, n = 40
$\bar{y}_{0}$	3583.66	2852.30	2303.78
$\bar{y}_{1}$	1691.43	1355.15	1087.35
$\bar{y}_{2}$	1690.47	1354.39	1086.73
$\bar{y}_{3}$	1490.62	1192.96	958.26
$\bar{y}_{4}$	1282.01	1024.46	824.15
$\bar{y}_{5}$	1278.03	1021.27	821.59
$\bar{y}_{6}$	1275.16	1018.37	819.03
$\bar{y}_{7}$	1274.17	1017.59	818.40
$\bar{y}_{8}$	1462.31	1181.19	950.65
$\bar{y}_{m}$	1136.84	921.83	748.56

**Table 2:** Percent relative efficiency (*PRE’s*) of the proposed estimator with the existing estimators

Estimators	Percent relative efficiency (*PRE’s*)		
	n′ = 60, n = 30	n′ = 65, n = 35	n′ = 70, n = 40
$\bar{y}_{0}$	100.00	100.00	100.00
$\bar{y}_{1}$	211.87	210.48	211.87
$\bar{y}_{2}$	211.99	210.60	211.99
$\bar{y}_{3}$	240.41	239.09	240.41
$\bar{y}_{4}$	279.54	278.42	279.54
$\bar{y}_{5}$	280.41	279.29	280.41
$\bar{y}_{6}$	281.04	280.09	281.28
$\bar{y}_{7}$	281.26	280.30	281.50
$\bar{y}_{8}$	244.79	303.05	376.54
$\bar{y}_{m}$	314.87	388.31	478.19
